# DICOMweb™: Background and Application of the Web Standard for Medical Imaging

**DOI:** 10.1007/s10278-018-0073-z

**Published:** 2018-05-10

**Authors:** Brad W. Genereaux, Donald K. Dennison, Kinson Ho, Robert Horn, Elliot Lewis Silver, Kevin O’Donnell, Charles E. Kahn

**Affiliations:** 1 Agfa HealthCare, 375 Hagey Boulevard, Waterloo, ON N2L 6R5 Canada; 2 Don K Dennison Solutions Inc., 205 Fern Cres, Waterloo, ON N2V 2P9 Canada; 3 Change Healthcare, 10711 Cambie Road, Richmond, BC V6X 3G5 Canada; 4 Fairhaven Technologies, Maynard, Massachusetts USA; 5 Canon Medical Research USA, Inc., 706 Deerpath Drive, Vernon Hills, IL 60061 USA; 6 Department of Radiology, University of Pennsylvania, Philadelphia, PA 19104 USA

**Keywords:** DICOMweb, DICOM, REST, Imaging, Interoperability, Standards, WADO

## Abstract

This paper describes why and how DICOM, the standard that has been the basis for medical imaging interoperability around the world for several decades, has been extended into a full web technology-based standard, DICOMweb. At the turn of the century, healthcare embraced information technology, which created new problems and new opportunities for the medical imaging industry; at the same time, web technologies matured and began serving other domains well. This paper describes DICOMweb, how it extended the DICOM standard, and how DICOMweb can be applied to problems facing healthcare applications to address workflow and the changing healthcare climate.

## Introduction

Medical imaging plays a critical role as a diagnostic tool. Reynolds concluded back in 2003, “while written descriptions of lesions and infectious processes may be detailed, the visual presentation of these conditions is much more accurate and effective for diagnosis and treatment” [1]. With that in mind, “image enablement” in healthcare software becomes paramount. Image enablement extends beyond inserting pictures into a health record; it includes the set of collaborative workflows that helps clinicians convey and interpret the patient’s condition in ways that text cannot. Because imaging is key to so many clinical workflows, if imaging exists, but is not accessible, clinicians may need to re-image the patient [2], which adds risk from ionizing radiation, creates unnecessary cost, causes delays in treatment, increases stress and discomfort for the patient and their families, and increases strain on hospital resources.

In order to address these challenges, all applications that touch upon medical imaging—be they Picture Archive and Communication Systems (PACS), Electronic Medical Records (EMR), Electronic Health Records (EHR), Radiology Information Systems (RIS), Vendor Neutral Archives (VNA), imaging acquisition devices, network gateways, and proxies—must agree to communicate information in a standard way and in a standard format.

The standard for medical imaging information is DICOM (Digital Imaging and Communication in Medicine). DICOM was first released in 1985, has undergone many changes, and has been adopted worldwide. The demands upon networking technologies have increased, which has brought about an evolution. Higher bandwidth wireless connectivity is now possible. At the same time, the devices clinicians use has changed, from a limited use of dedicated workstations to expanded use coupled with mobile devices with limited bandwidth, limited resolution, and limited battery life. The way information is accessed has changed, from being limited within a hospital to being shared across the enterprise and between enterprises. These changes necessitated new security requirements and enterprise IT policies for DICOM. As a result, DICOM adopted new technologies beginning in 2003. DICOM introduced web technologies with the inclusion of WADO (Web Access to DICOM Persistent Objects). Those DICOM web services have evolved to become the standard called DICOMweb.

## Review

The most basic imaging use case includes scheduling, acquiring, managing, processing, displaying, reporting on, and distributing images. A patient is scheduled for a diagnostic exam (e.g., a chest radiograph). A radiography technician uses an imaging device (e.g., an X-ray unit) to acquire images and transmit them to an archive (e.g., a PACS). A radiologist accesses these images, interprets them, and creates a report that is sent to the Radiology Information System (RIS) for distribution to the referring physician, sometimes alongside the images. During interpretation, radiologists may need to see prior imaging studies. Increasingly, such studies are available from other institutions, from jurisdictional, regional or national archives, from cloud-based image sharing solutions, or brought by patients or their families on a CD or DVD. Today, one may choose to store imaging information to a long-term archive, burn the images to a CD or DVD for the patient, share it through an EHR, render them on mobile devices (like tablets or phones), digitally reconstruct the images to form 3D visualization, and share them to portals (for ordering physicians or to patients themselves).

DICOM is the standard for retrieving, storing, printing, and transmitting information in medical imaging, and includes both a file structure and communication protocol [3]. It is an international standard (ISO 12052) for medical imaging and related information. DICOM defines formats for images, waveforms, and derived structured data to provide the entire dataset necessary for clinical use. It enables imaging department workflow management, media exchange and printing, and it achieves this using service-based network protocols over TCP/IP and HTTP. DICOM defines how to store acquired images from modalities such as CT, MRI, X-Ray, Ultrasound, Angiography, PET, microscopes, cameras and ophthalmological devices. These image instances may be formatted as single or multi-frame objects, multi-dimensional volumes, or cine loops. These may be original or derived images and may be associated with annotations and other presentation settings. These objects can refer to related documents, such as requisitions, consent forms, and reports. Other non-image medical data, like ECG waveforms, ultrasound measurements, or radiation dose information, can also be stored using DICOM. For all objects, DICOM defines significant meta-data, for example, for patient identification and demographics, ordering information, acquisition parameters, and workflow context. This metadata is used to query, sort, route, display, and manage the images.

**Fig. 1:**
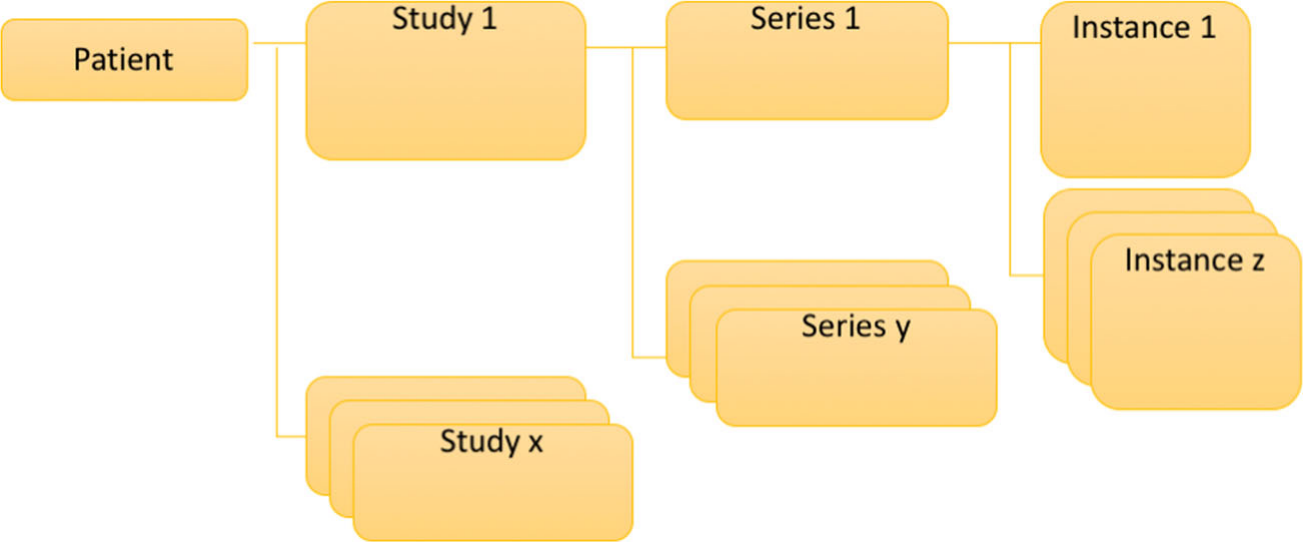
DICOM information model

In terms of an information model, DICOM defines a logical hierarchy, starting with the patient, as demonstrated in Fig. 1.

- A patient represents the human or animal subject of medical imaging and has a collection of imaging studies.
- Each study represents an organized set of images for a particular diagnostic purpose and contains a collection of series.
- Each series represents a single acquisition event on a single machine and contains a collection of instances.
- Each instance represents a single data object which may be an image frame, a set of images frames, or a document (measurements or a report).

Furthermore, even when distributed or accessed independently, each DICOM instance is fully usable since it is fully self-describing. Each instance contains a complete set of metadata, including:

- Study-level attributes, such as the study unique identifier, the study description, and the patient demographics.
- Series-level attributes, such as the series unique identifier, the modality, and the body part imaged.
- Instance-level attributes, such as the instance unique identifier, the image resolution, and the table position where the image was acquired.

Each attribute of the metadata has a defined data type and cardinality and is identified by a 32-bit tag—usually shown as two sets of four hexadecimal digits. For example, the tag (0010, 0010) identifies the data attribute containing the patient’s name, which has the datatype of a person name, and can occur at least once. Each DICOM instance is identified by a globally unique identifier (meaning, no two instances ever use the same identifier, worldwide).

DICOM also defines non-image objects that are associated with images and studies—these include:

- Key object selections (called KOS) identify one or more images intended for a specific purpose, such as representing something clinically significant.
- Grayscale presentation states (called GSPS) describe how an individual image or a set of images can be annotated, rotated, cropped, and brightness and contrast adjusted, without modifying the underlying image instance.
- Structured reports (called SR) encode measurement data in a machine-readable way (e.g., radiation dose SR for ionizing radiation exposures)
- Encapsulation objects take non-DICOM files such as CDA [4] (clinical document architecture), PDFs, or textual reports and store them in the DICOM format so they can be stored and managed as part of an imaging study.

As new imaging-related technologies emerge, such as multi-energy CT, or clinical 3D printing, new object types are defined.

To understand how DICOMweb became part of the standard, it is important to understand the healthcare and technology ecosystems. Technology in general, and specifically in healthcare, had evolved and so the technologies used in medical imaging had to evolve as well to meet the needs of clinical systems utilizing imaging. Imaging is not the major focus for many healthcare systems, which thus may be reluctant to invest time and effort into understanding a “niche” protocol such as DICOM. There may be safety risks if systems are misconfigured or misused; for example, displaying an arm X-ray for an amputation surgery without the laterality markers specified in the DICOM metadata can lead to serious errors. There are many nuances associated with display of medical images, including tag morphing, lifecycle management, scaling, and dealing with huge data sets. In addition, given that Information Technology (IT) personnel are familiar with concepts such as image display and content upload over the web, and data parsing, historically, DICOM did not use the same technology commonly encountered for those applications. The DICOM file format is not an image format recognized by internet browsers (unlike JPEG, GIF, PNG, and others). Storing newly acquired images (such as, from a clinician’s smartphone for an ad-hoc acquisition) requires in-depth knowledge of both the acquisition parameters and DICOM rules to create the right tags and file format. Managing the image lifecycle (from acquisition, to ingestion, to storage) requires specialized software. Metadata extraction requires parsing binary data, as opposed to XML or JSON technologies. Scaling a system upward requires specialized knowledge of imaging systems, unlike growing a set of clustered nodes in a virtual cloud infrastructure. Storage and bandwidth/latency issues are magnified as image data sets grow into gigabytes and terabytes. There was a need to leverage new technologies in medical imaging, and the users and vendors in the medical imaging industry needed ways to take advantage of web technologies in ways that were compatible with their significant investments in existing infrastructure.

Aside from the technology, the needs of clinicians and healthcare institutions changed over time too. Patient-centered care continues to evolve and it demands consistent access to data wherever it is stored (including other institutions) from anywhere the clinician happens to be (in the hospital, at home, in an airport terminal) and on whatever device the clinician is using (a desktop computer, a tablet, a telemedicine cart, a patient bedside terminal, a hallway-mounted computer, or a mobile phone). Hospital IT departments have evolved from individual departmental specialists into enterprise teams, marked with some associated loss of specialist knowledge. Application development paradigms have shifted toward service-oriented architectures. Security and privacy concerns have become much more prevalent, especially with moves to cloud services that have begun to reflect patient confidentiality regulations and protections. The era of “pushing” healthcare data from system to system has started to come to an end, and the era of “pulling” data on demand has begun. Furthermore, the principles of traditional radiological imaging are being supplanted by a broader concept of enterprise imaging with governance of all clinical images regardless of their origin. Enterprise imaging is “a set of strategies, initiatives and workflows implemented across a healthcare enterprise to consistently and optimally capture, index, manage, store, distribute, view, exchange, and analyze all clinical imaging and multimedia content to enhance the electronic health record.” [5] So, for both technology and non-technology reasons, a different approach had to be taken to address the needs of users and systems across the spectrum.

Starting in 2003, a web-enabled DICOM extension began to take shape. Initially called WADO, or Web Access to DICOM Persistent Objects, it defined a method to retrieve DICOM objects using HTTP, the same technology used on the web. Over time, other services were added to DICOM to augment the retrieval use case—this included search via QIDO-RS (Query based on ID for DICOM Objects) and upload via STOW-RS (Store over the Web). WADO was upgraded to WADO-RS to more closely follow the emerging RESTful style of web interfaces. Collectively, these technologies became known as DICOMweb. DICOMweb adds HTTP powered services to DICOM, modeled after the style of Representational State Transfer (REST). REST has become the dominant API style used by companies and market verticals all around the world [6]. REST enables systems that are scalable, fault-tolerant, recoverable, secure, and loosely-coupled [7]. DICOMweb was designed to augment existing DICOM systems with web technologies. This is important because, given the penetration of DICOM-enabled systems all around the world by hundreds of companies producing trillions of DICOM images, it is not feasible to switch to a completely different standard without disruption. DICOMweb makes it possible to render DICOM instances into widely used consumer-friendly formats suitable for web browsers on both desktops and mobile devices, without requiring a mass conversion of the existing historic data or information model. DICOMweb preserves the historical DICOM information model and metadata rules while enabling access to this information in web formats.

Through embracing web technologies, DICOMweb helps to enable communication of imaging data not only to systems within an institution but also to those outside. Further, it supports the security necessary to make this safe natively using the same industry standards that secure commercial activities like banking. Industry-standard security and acceleration appliances can be used to achieve greater performance, since they are optimized for industry-standard HTTP traffic. And, through using RESTful principles, DICOMweb allows for rapid development by software developers. It is possible to access basic DICOMweb retrieval services directly in a browser with no special tools needed; to access traditional DICOM services, often specialized software libraries were needed.

Medical imaging over the web has undergone its own evolution, as well. This technology evolution is indicated by the suffix on the service names; “URI” representing a Uniform Reference Identifier method, “WS” representing a Web Service (or SOAP) method, and “RS” representing a RESTful method. The original WADO service, now known as WADO-URI, was developed to provide retrieval of DICOM instances using query parameters on a single resource. For example, a WADO-URI service resource URL may look like “https://myserver.com/wado?studyUID=2.16.840.1.1.2.3&seriesUID=2.16.840.1.4.5.6&objectUID=2.16.840.1.7.8.9” where the resource is named “wado” and the parameters passed include “studyUID”, “seriesUID”, and “instanceUID”. This has since evolved into what is known as WADO-RS, which uses multiple resource concepts to provide retrieval services. For example, “https://myserver.com/studies/2.16.840.1.1.2.3/series/2.16.840.1.4.5.6/instances/2.16.840.1.7.8.9” refers to a hierarchal relationship of resources—the 2.16.840.1.7.8.9 instance resource in the “instances” resource in the “2.16.840.1.4.5.6 series resource in the “series” resource in the 2.16.840.1.1.2.3 study resource in the “studies” resource. This more accurately reflects how the study contents are nested and provides the ability to request bundles of resources at the series or study level. DICOMweb also aligns with evolutions in other healthcare APIs; an example of this is HL7’s Fast Healthcare Interoperability Resources (FHIR) [8]. Reflecting evolving approaches to network communications, for a period DICOM also defined a web API called WADO-WS using the SOAP (Simple Object Access Protocol) specification, but this API has since been retired from the DICOM Standard [9]. During the same time period, an independent group began to work on a competing standards project, called MINT (Medical Imaging Network Transport) [10]. Many of the concepts from MINT were later re-worked and adopted into the development of the DICOMweb *-RS services, with input from the user and vendor communities alike.

## Discussion

**Fig. 2:**
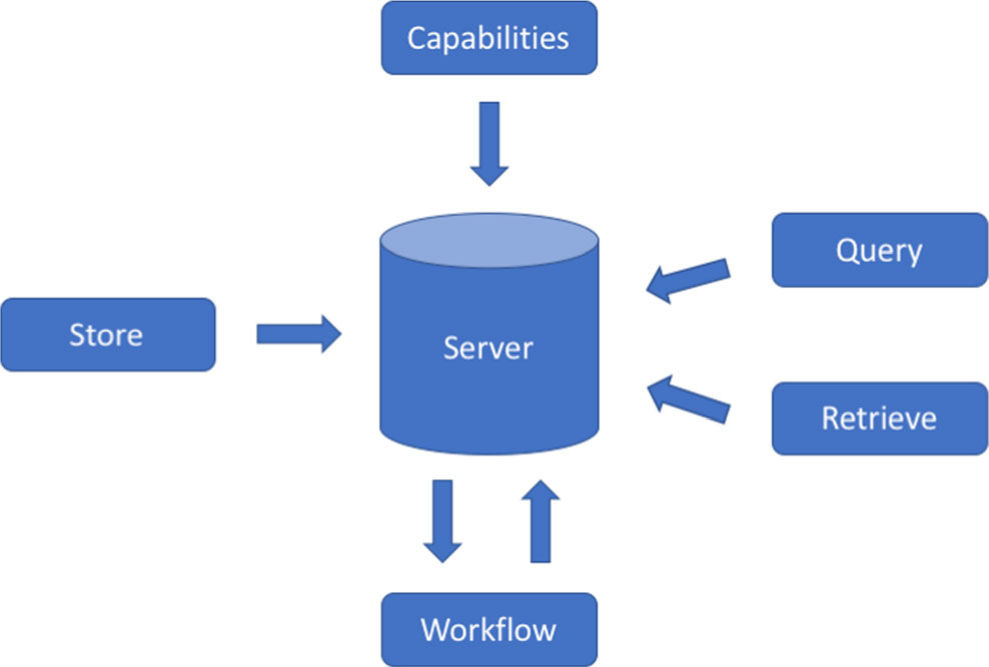
DICOMweb operations

DICOMweb defines five core services that correspond to traditional DICOM operations—query, retrieve, store, workflow, and service discovery (see Fig. 2).

Part 18 of the DICOM Standard defines these services using a common language and terminology. DICOMweb defines resources and operations on those resources using the style of REST on top of the HTTP standard. Studies, series, and instances, along with their identifiers, are all resources that can be retrieved (via GET operations) and created (via POST operations).

Query operations, referred to as QIDO-RS (Query based on ID for DICOM Objects using RESTful Services), are invoked by requesting one of the generic resources (e.g., “https://myserver.com/studies/2.16.840.1.1.2.3/series/2.16.840.1.4.5.6/instances” requests the “instances” resource for a specific study and series). Additional request parameters are typically supplied to filter the results. For example, “GET http://server.com/studies/?00100010=DOE^JOHN” will return all studies that belong to patients named John Doe. Responses can be returned in either XML or JSON format, depending on what the client requests and what the server supports.

Retrieve operations, referred to as WADO-RS (Web Access to DICOM Persistent Objects using RESTful Services), are invoked by requesting one of the specific identifier resources (e.g., “https://myserver.com/studies/2.16.840.1.1.2.3/series/2.16.840.1.4.5.6/instances/2.16.840.1.7.8.9” requests the instance with the unique identifier “2.16.840.1.7.8.9”). Without further request parameters, this will return a full DICOM instance—a binary object that contain the metadata payload and pixel data. At an instance resource level, only one object is returned, but at the study or series resource level—for example, “https://myserver.com/studies/2.16.840.1.1.2.3/”—all of the instances in all of the series of that study are returned. Rendered images suitable for display in browsers can also be requested by specifying the media type, like “image/jpeg” (a JPEG image) or “image/png” (a PNG encoded image). Images can be requested to be a particular size or to be cropped to contain only a specific region of an image or to have a presentation state applied.

Store operations, referred to as STOW-RS (Store over the Web using RESTful Services), are invoked to create instances by uploading instances to the “study” resource (e.g., “https://myserver.com/studies”) with pixel data and a properly formed set of metadata in either XML or JSON. A set of instances can be uploaded as well. In response, the server returns a receipt of what was processed, and what failed processing. With the store operation, an application is able to easily take a standard JPEG image (by a consumer camera or mobile phone, for example) and upload it to a DICOM archive with an HTTP POST with a few key metadata elements in XML.

Workflow operations, referred to as UPS-RS (Unified Procedure Step using RESTful Services), are invoked by creating, claiming, and updating specific work item resources that store and communicate the state of a work item, its inputs and outputs. Systems can create work items for other systems to fulfill and monitor their progression through to completion. Furthermore, systems can subscribe to receive notifications whenever a work item is updated. The notifications use web sockets [11] (defined as part of the HTTP protocol).

Service discovery operations are invoked by using the HTTP OPTIONS verb [12] on any resource. This allows client systems to find out in a machine understandable way what DICOMweb services are available on a given server. This is particularly useful since not all systems implement all DICOMweb services. DICOMweb servers return a document that defines what the host server is capable of, using a description language called WADL (web application description language).

For DICOM instances that are not in the patient-study-series-instance data structure, there is also a non-patient object service. This includes, for example, color profiles used in the rendering of images, as well as CT protocols used when acquiring images.

For the convenience of developers, these DICOMweb services can return metadata rendered using Extensible Markup Language (XML) [13], or JavaScript Notation (JSON) [14]. Both indicate the relevant DICOM tag, the data type, and the specific value of that field. Consider a study date of April 9, 2013 contained in DICOM attribute [0008,0020] which has a DATETIME value representation.

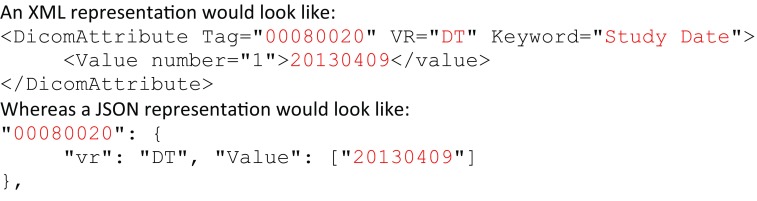



Data that is unwieldy to return in a response (such as binary content) can be replaced in the response by a bulk data reference, which can be separately retrieved if the client needs to do so.

## Summary

In summary, DICOMweb, and DICOM as a whole, represents medical imaging in a standard way allowing products around the world from hundreds of vendors to interoperate successfully in all sorts of use cases. DICOMweb enables collaborative use of medical imaging in medical use cases combining imaging and non-imaging data. When presenting a report of findings in a health application, there was traditionally just text, perhaps organized in sections. By leveraging DICOMweb, reports can contain links to original images, derived images, and supportive data. Using DICOMweb, those links allow the images to be retrieved in a displayable format and presented inside the health application.

DICOMweb is now a stable standard. New applications of DICOMweb can enable next generation technologies, such as image analytics and machine learning, by lowering the barrier to entry to retrieve instances appropriate for analysis. By preserving the information model, it is entirely tractable to create a proxy system that could add DICOMweb interfaces to an existing DICOM product that has not implemented the DICOMweb components. Furthermore, DICOMweb enhancements are being added; for example, in 2017, new resources at the study, series and instances levels is being developed to return small representational images (e.g., “thumbnails”) suitable for display in applications to aid clinicians in selecting the data they wish to display.

DICOMweb provides methods that are common across every market vertical and sector. It provides the same API methodologies and strategies that are familiar to software developers. It lowers the barrier to entry of working with medical images by using familiar tools. DICOMweb enables patient-centric care by providing secured and protected modern web-driven architectures that are both backward-compatible and forward-thinking. Improving access to imaging and reducing the cost of interoperability makes imaging more ubiquitous; this in turn reduces delays and repeat examinations and enables clinicians to better collaborate with care teams locally and globally, ultimately improving access to quality care.
